# Biomimetic Shading Systems: Integrating Motorised and Moisture-Responsive Actuation for Adaptive Façades

**DOI:** 10.3390/biomimetics10100711

**Published:** 2025-10-20

**Authors:** Negin Imani, Marie-Joo Le Guen, Nathaniel Bedggood, Caelum Betteridge, Christian Gauss, Maxime Barbier

**Affiliations:** 1 Bodeker Scientific, Wellington 6012, New Zealand; 2 Centre for Sustainability, University of Otago, Dunedin 9016, New Zealand; ngbedggood@gmail.com; 3 Open Polytechnic, Lower Hutt 5011, New Zealand; 4 Scion Group, New Zealand Institute for Bioeconomy Science Ltd., Rotorua 3046, New Zealand; mariejoo.leguen@scionresearch.com (M.-J.L.G.); caelum.betteridge@scionresearch.com (C.B.); maximebbr@gmail.com (M.B.); 5 School of Engineering, University of Waikato, Hamilton 3240, New Zealand; christian.gauss@waikato.ac.nz

**Keywords:** biomimetic, adaptive building façade, façade technology, automated shading devices, 3D printing, biocomposite, energy efficiency, automation

## Abstract

A biomimetic adaptive façade applies natural principles to building design using shading devices that dynamically respond to environmental changes, enhancing daylight, thermal comfort, and energy efficiency. While motorised systems offer precision through sensors and mechanical actuation, they consume energy and are complex. In contrast, passively actuated systems use smart materials that respond to environmental stimuli, offering simpler and more sustainable operation, but often lack responsiveness to dynamic conditions. This study explores a sequential approach by initially developing motorised shading concepts before transitioning to a passive actuation strategy. In the first phase, nine mechanically actuated shading device concepts were designed, inspired by the opening and closing behaviour of plant stomata, and evaluated on structural robustness, actuation efficiency, ease of installation, and visual integration. One concept was selected for further development. In the second phase, a biocomposite made of polylactic acid (PLA) and regenerated cellulose fibres was used for Fused Deposition Modelling (FDM) to fabricate 3D-printed modules with passive, moisture-responsive actuation. The modules underwent environmental testing, demonstrating repeatable shape changes in response to heat and moisture. Moisture application increased the range of motion, and heating led to flap closure as water evaporated. Reinforcement and layering strategies were also explored to optimise movement and minimise unwanted deformation, highlighting the material’s potential for sustainable, responsive façade systems.

## 1. Introduction

Building operation accounts for 30% of global energy consumption [1] and 27% of energy sector emissions [2]. As the impacts of climate change intensify, reducing energy consumption in buildings has become an urgent priority. Evidence suggests that adaptive façades can reduce energy consumption, improve thermal performance, and enhance occupant well-being.

Conventional materials are failing to respond to our changing climate, as evidenced by the International Energy Agency’s 2022 report calling for higher-performance building façades. This while responsive facades outperform traditional solutions, achieving up to 65% higher efficiency [3].

Biomimetic Adaptive Building Façades (Bio-ABFs) are inspired by plants’ adaptation to changing weather and can mitigate operational energy use while enhancing occupants’ comfort [4,5,6,7,8,9,10,11]. The existing adaptive facades that operate at a real scale are motorised and use mechanical equipment for activation. The research shows that maintenance remains a critical challenge for such responsive facades.

The Institut du Monde Arabe (1981–1987) featured a complex system of 240 motor-controlled apertures, but due to high maintenance demands, it gradually failed, becoming non-functional within six years [12]. Similarly, the GSW Headquarters (1999) employs a double-skin façade with actuated blinds, which, while still operational after 25 years, requires more maintenance than conventional buildings [13]. Other examples, such as the Kiefer Technik Showroom (2007) and St. Ingbert Town Hall (2010) [14], also struggle with maintenance-intensive, bespoke systems. To ensure long-term viability, adaptive façades must be designed for durability, ease of maintenance, and standardisation.

While several studies have been conducted on kinetic façade systems (Table 1), there remains a lack of focus on the kinematic design of individual façade modules (shading systems) and how they can influence façade operation when integrated into the façade. A more holistic approach is required, one that considers the existing challenges associated with motorised systems. Although it is not an easy task to develop a systematic design framework for developing façade shading systems, it is possible to evaluate the kinematics of shading modules concerning the practical challenges they pose when integrated into the building envelope. 

As identified by [5] when translating plant thermoregulation strategies into architectural equivalents, it is essential to consider the thermoregulation variables present in the heat transfer equation. A similar approach in architecture could yield comparable results. Interestingly, regardless of the specific thermoregulation solution used by plants, the main variables in thermoregulatory mechanisms and the heat transfer equation include either temperature gradient, solar heat coefficient (absorption or transmission), surface area, and heat transfer coefficient. These variables can be categorised under either spatial or morphological principles. This insight highlights the need to shift the biomimetic design approach from a focus on form to one centred on function. It is not the form of the plant organs or the specifics of their observed kinetics that directly impact heat transfer, but rather that these mechanisms ultimately depend on the fundamental principles and variables outlined above. That said, in the design of shading systems, the primary focus should not be on replicating the morphological forms or kinematic movements observed in biological analogues. Rather, emphasis should be placed on the underlying principles of these systems in thermal adaptation. The form and kinematics of the design of shading systems are significant only to the extent that they facilitate construction and operational efficiency, including the ease of fabrication, automation, maintenance, durability, and seamless integration within the building’s broader management framework.

Existing Bio-ABFs rely on expensive, high-maintenance mechanical systems for adaptive façade geometries [8,10,15,16,17,18,19] and smart material responses attached to moving elements of the shading device [20,21,22,23,24,25,26,27]. 4D printing with biomaterials offers more cost-effective solutions, enabling the creation of dynamic, multifunctional structures [28,29].

**Table 1:** Synthesis of kinetic façade systems uses and findings in buildings drawn by past studies.

Building	Year	Prototype Scale	Fabrication Tool	Shape Change	Material of the Adaptive Component	Regulation	Design	Software Used	Authors
**Buckling system**	2022			Buckling, electricity	Shape Memory alloy.	Light	Bistable mechanism	Finite element analysis (FEA)	[30]
**Microalgae bio-reactive** **facade**	2022	Small scale	Lab tools	Algae growth, mechanical component	Algae	Light & heat	Control of bioreactor (microalgae medium flow)	-	[31]
**Kinetic biomimetic Facade**	2022	Small scale	Punch press industrial tools	Difference in the coefficient of expansion, temperature change of the material	Thermobimetal	Light & heat	Flowerlike triangular petals Open & close mechanism	Rhino, grasshopper. Finite element analysis (FEA)	[10]
**La Seine Musicale**	2017	Full scale- France		Pneumatic system mounted on a rail	Solar Panels	Energy	Honeycomb structure & Trussed steel framework		[32]
**Ocean Pavilion**	2012	Full scale	Labour work	Torsional buckling, mechanical actuator	Laminated sheet. glass fibre reinforced polymer (GFRP)	Light	Analog control of a mechanical System + buckling	-	[33]
**Dancing Pavilion**	2016	Full scale-Rio, Brazil	-	Swivelling metal panels, sensors	Metal disc controlled by a stepper Motor	-	-	Animation software	[32]
***Strelitzia reginae*-Flctofin**	2015	Single-component prototype	Labor fabrication	Punctually applied load, mechanical control	Glass fibre reinforced polymer (GFRP)	Heat & light	Rectangular frames	FAM, CAD	[34]
**Hugroskin**	2014	Full scale	7-axis industrial robot to CNC mill, vacuum moulding	Anisotropic structural and Hygroscopic characteristics of wood, ambient humidity	Elastically bent plywood. Wood veneers	Air flow	Triangular shape	Finite element analysis (FEA)	[35]
**Al-Bahr Tower**	2012	Full scale	-	Linear actuators, building management System	Stainless steel supporting frame, aluminium dynamic frame, polytetrafluoroethylene (PTFE) coated Fibreglass panels	Light	Honeycomb-inspired structure for the curtain wall. Triangular unit for kinetic structure	CATIA Tool, Rhino Scripts integrated into BIM	[36]
**MEDIA-ICT-Building**	2011	Ful scale-Barcelona	Laser-cut machine	Pneumatic system, Lux meters trigger Of pneumatic shading or fog system	ETFE (ethylene Tetrafluoroethylene)	Light & temperature	Lattice structure	Finite element analysis (FEA)	[37]
**Oval Cologne Offices**	2010	Full Scale—Germany	-	Sensors	Glass	Heat & light	Rectangular frames	-	[38]
**Ingbert Town Hall**	2009	Full scale-Germany	-	Motorised pushing rod	Electropolished expanded stainless Steel sheet	Light	-	-	[14]
**Milsertor centre**	2008	Full-scale Austria		18 Electrical motor, rail and gliding rod, programmed System	Plexiglass panel	Light	Rectangular frame		[14]
**Kiefer Technic Showroom**	2007	Full-scale Germany	-	Electrical motor, rail and roller, a programmed System	Aluminium panel	Light	Rectangular frame	Programmable BUS/PLC system.	[39]

The development of 4D-printed building materials can enhance architectural design freedom and increase the environmental efficiency of adaptive façades [40]. The long-term benefits of these preprogrammed materials could include self-assembly and self-repair capabilities and addressing current technical challenges such as high maintenance and reliance on complex computer-controlled systems. 

Early experiments in this field focused on harnessing the material properties of various metals to enable movement in response to temperature changes. Initially, research centred on shape memory alloys (SMA) [41]. However, due to their high cost and low mechanical strength, alternative materials had to be explored [42]. This led to increased interest in bimetallic systems over the past decade. These systems consist of rigidly connected metal sheets with differing thermal expansion coefficients [43]. When heated, one sheet expands more than the other, causing the structure to bend in one direction; as it cools, it returns to its original shape.

Sung [43] leveraged these properties to develop innovative systems for ventilation and shading. One example is the Bloom pavilion, composed of 414 stacked, hyperbolic paraboloid-shaped bimetallic panels. These panels automatically open at a specific temperature to release excess heat and close when cooled to retain warmth. Another system Sung [43] developed was the Pivot Shade System, which integrates bimetallic strips into a double-skinned façade to function as a shading device. Similar to the GSW Headquarters, these strips bend when heated, adjusting their angle to provide optimal shading for the building’s interior. Collectively, these examples demonstrate the potential of bimetallic systems to regulate indoor temperature, shading, and ventilation while offering greater durability than traditional adaptive systems.

Inspired by flower petals Charpentier, Cruz [10] explored the use of bimetallic systems at a more advanced level. The rigid sections of the petals incorporated photovoltaic panels to generate passive energy while mitigating solar gain. Despite these advancements, the use of bimetallic panels was not considered in later development stages. Although bimetallic materials provide increased rigidity compared to other shape-changing materials, their high cost, limited availability, and lack of recyclability make them less viable for large-scale adoption [10]. These factors present a significant barrier to the widespread use of bimetallic systems in adaptive architecture. While they offer durability and robustness, their expensive and complex manufacturing process hinders their practicality.

Other similar projects have used layered copper and polyethene. However, due to cost considerations, these materials were replaced by cross-laminated timber veneer, taking advantage of the difference in expansion rates of timber running parallel and perpendicular to the grain. Coating was also used to modify the expansion speed [44]. 

The HygroSkin pavilion utilised the hygromorphic properties of timber. Developed by Achim Menges with the Institute for Computational Design at the University of Stuttgart, the pavilion responded to humidity changes, with timber absorbing moisture and expanding to actuate shutters constructed from veneer layers [35]. A different system developed by Mazzucchelli, Alston [45] integrated thin-film solar cells with a hygromorphic layer composed of two wooden slats from different wood types and trunk cuts. The deformation resulted from the differing expansion coefficients of the two wooden slats, causing them to expand and contract at different rates in response to temperature and humidity changes. 

Achieving shape-changing capabilities is not restricted to architecture and has already been demonstrated in various other fields during the last decade. Table 2 compares examples of various shape morphing 4D printed composites, including PLA, chitosan-based polymers, hydrogels, soybean oil-based polymers, wood-fill filaments, and bacterial films, based on their stimuli responses, technical characteristics, fabrication methods, and applications. These materials respond to heat, pH, moisture, light, and humidity. Fabricated mainly through 3D printing, inkjet, or laser techniques, their transition times range from immediate to hours. Applications span biomedical fields (drug delivery, scaffolds, adaptive interfaces), engineering (solar tracking, filtration, acoustic wave control), and soft robotics, demonstrating their potential in self-morphing structures, responsive surfaces, and bio-compatible innovations.

While most research on adaptive building systems has focused on either passive or active actuation strategies, there remains a knowledge gap in understanding how these approaches can be effectively integrated. In this context, passive actuation refers specifically to the intrinsic shape-changing behaviour of the shading component itself—excluding supporting mechanisms such as hinges or linkages, which have already been widely studied. A sequential approach that combines passive and active strategies offers a promising pathway to overcome current limitations, enabling façade systems that are both energy-efficient and highly responsive. Advancing this sequential model is essential to improve the performance, reliability, and scalability of next-generation climate-adaptive building technologies.

While kinetic façade systems have been extensively explored, few studies have addressed the practical integration of motorised shading systems into building envelopes from a kinematic design perspective. Many instead focus on biomimetic movements or forms. As discussed earlier, this research takes a more structured approach, aiming to develop and evaluate formal and kinematic solutions that directly address the construction and operational challenges of kinetic adaptive façades. The research builds on existing work in modularity, customisation, and active automation within solar shading systems integrated into Bio-ABFs.

The proposed design components are inspired by stomatal regulation in plants, where opening and closure control water exchange and thermal balance, serving as a direct analogy for the adaptive shading response.

## 2. Materials and Methods

### 2.1. Design Development of Motorised Shading Systems with Mechanical Actuation (Using 3D Printing Prototyping)

The key criteria for the development process were chosen based on the core engineering principles of product development to ensure a practical outcome. Each judging criterion balances the principles of functionality (Actuation, Occlusion), longevity (Simplicity and Robustness, Repairability), practicality (Mounting) and also with the crucial element of form (Design Appeal) [55]. 

Our evaluation criteria are supported by prior studies: ease of maintenance and repairability [56,57], mounting feasibility [58], actuation method [57], practicality [58] occlusion ratio [59,60], design simplicity [7,60,61], durability and robustness [62] have all been identified as key considerations in façade and shading system design.

(1) Simplicity and Robustness

Minimise the number of joints and moving parts;

Avoid overly complex slats, plates, or geometries that could compromise reliability.

(2) Actuation Method

Evaluate the suitability of servos versus linear actuators;

Consider practical methods for mounting and integration.

(3) Repairability

Explore design strategies that allow for easy maintenance and future repairs.

(4) Mounting Method

Assess the feasibility of mounting the device on buildings, both in retrofits and new constructions;

Minimise interference from the structural frame or mounting points.

(5) Occlusion Ratio

Aim for a dynamic range of 0% to 100% occlusion between fully open and closed states.

(6) Design Appeal

Ensure the design not only performs its function but also complements the architectural aesthetics;

Consider the visual impact of the device when repeated in a façade grid, will it appear cohesive or visually cluttered?

**Figure 1:**
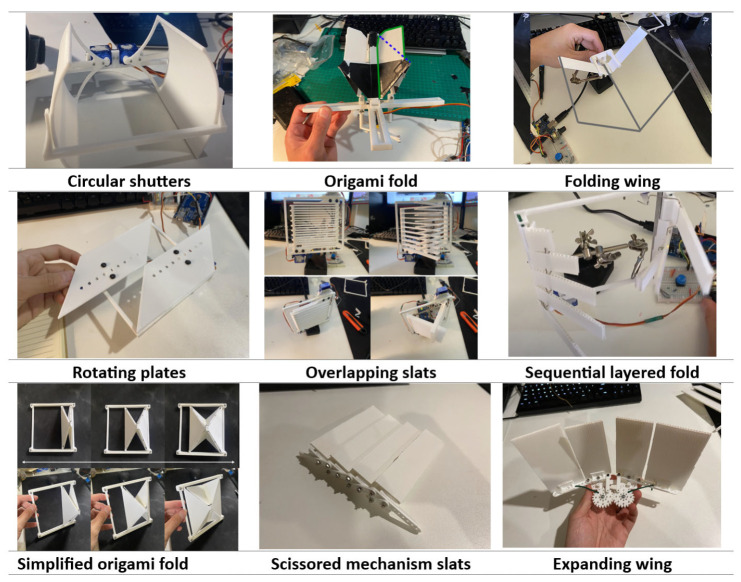
Design concepts for adaptive façade shading systems.

A range of concepts was developed, each demonstrating distinct mechanical behaviours, aesthetic qualities, and feasibility constraints. The following is a summarised overview of nine design concepts that were explored during the design process (Figure 1):

**Circular Shutters:** This concept consisted of two quarter-cylinder panels that rotated to form a semicircular closure. Each panel was rotated using two servos, although a single motor with interlinked gears could also have been used. A key limitation was the depth required within the frame to house the retracted shutters.

**Origami Fold:** Inspired by early origami folds, this design introduced complex motion through multiple hinges and moving parts. Two corners of the square panel travelled along a linear rail, while the other two remained free-floating. Actuation was considered via a linear actuator or a motor with linkages. A variation of the original origami design, this version modified the panel shapes to influence the system’s overall form. In the closed position, the arrangement resembled a tulip, demonstrating how geometric changes could affect visual outcome.

**Folding Wing:** This concept used a compact linear actuation system to drive a larger folding movement. A central hinged section moved along a straight path, while the side panels were guided by rotating points. Although simple to actuate and mount, the design required further optimisation to reduce frame depth and improve occlusion performance.

**Rotating Plates:** This design used two flat shutters that rotated via dual servos. It investigated whether overlapping shutters that extended into adjacent modules could conceal the structural frame beneath, aiming to produce a more seamless appearance across the façade. This variation repositioned the rotating plates to alter their overlap behaviour. While lateral overlap with neighbouring modules was retained, vertical integration was reduced. Making the plates removable was also considered to facilitate maintenance and support a variety of material finishes.

**Overlapping Slats:** This design featured slats actuated by two servos, creating a phasing visual effect as they moved past one another. Although not fully opaque in the closed state, the design effectively blocked sunlight at common angles. As the slats transitioned between states, they formed a dynamic zig-zag texture across the surface.

**Sequential Layered Fold:** This concept was built upon the use of two servos to produce a cascading movement. The first slat was designed to move initially, then hook onto the next one, initiating a sequence that created a fanning effect. While actuation was simple, the slats needed to be robust and effective at varying angles, which introduced further design challenges.

**Simplified Origami Fold:** This design simplified the original origami-inspired concept by reducing the number of hinges. It retained the key folding motion while offering easier fabrication and a straightforward linear actuation path.

**Scissored Mechanism Slats:** his concept involved slats attached to a scissor mechanism. Although it occupied significant space when compressed, it allowed light penetration through controlled gaps. The design potentially shared actuation components with other linear rail systems and benefited from established mechanical principles.

**Expanding Wing:** This design employed gears and linkages to produce a single, smooth folding motion. The plates stretched and retracted to reveal or conceal the façade. While it was the most visually dynamic, it was also the most complex to implement as a full-scale, robust system.

Across these 9 concepts (Figure 1), most shared two primary actuation strategies: (1) rotational via dual motors/servos, or (2) linear via actuators on a central rail. These shared mechanisms suggest potential for standardised frames supporting multiple design types, though this could complicate frame design due to differing spatial and mechanical demands.

**Figure 2:**
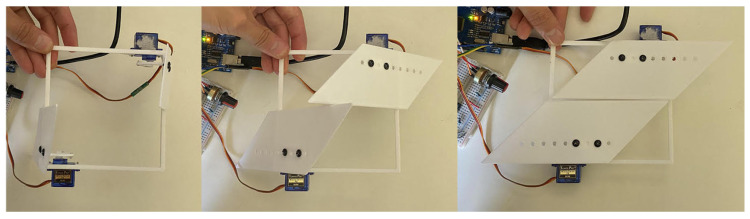
Rotating plates kinematic design concept.

Based on six key criteria and following a comparative analysis of the nine design concepts (Results and Discussion section), three concepts were shortlisted, with the concept ‘Rotating Plates’ ultimately selected for further development (Figure 2). This design was subsequently used for 4D printing in the second phase of the research.

**Figure 3:**
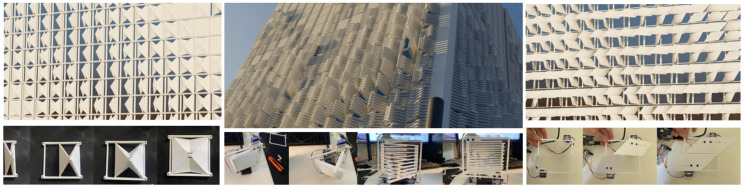
Design alternatives: Simplified Origami Fold, Overlapping Slats, and Rotating Plates from left to right.

The three shortlisted design concepts were modelled and rendered in Blender to visualise the scale of each module in the context of a human and a building (Figure 3). It was determined that each module would be sized at 800 mm by 800 mm, with advanced prototyping to be conducted at a 1:5 scale. 

To determine the appropriate size of the prototypes, two main aspects had to be considered: first, to build the prototypes to a scale that could realistically represent a full-size building and produce meaningful data; and second, to serve as an interactive display to help communicate the design vision. The 1:5 scale was a practical choice because it balanced the limits of the 9 g servos, which could move the 3D-printed parts without stalling, and allowed enough modules to be built and tested. 

PLA was selected for the fabrication of motorized systems due to its excellent combination of printability, mechanical performance, and environmental safety. The eSUN Matte PLA+ used offered high rigidity, low shrinkage, minimal warping, and a lighter weight than standard PLA, reducing both material cost and overall mass while remaining durable and easy to process.

### 2.2. Design Development of Non-Motorised Shading Systems with Passive Actuation Based on 4D Printing

**Figure 4:**
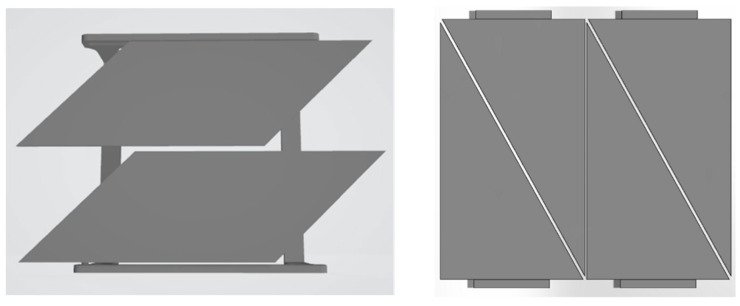
Initial design concept (**left**); modified design concept as assembly (**right**).

Building upon this design the parallel step was to investigate the addition of a passive actuation based on the materials composition. The actuated design concept comprises two plates mounted on a fixed frame (Figure 4 left). The opening mechanism of the flaps happens in response to variation in water content and temperature. The design of the shading device was simplified by dividing each flap into two triangular surfaces to enhance control over monitoring the shape-change mechanisms of individual flaps (Figure 4 right). 

#### 2.2.1. Compounding and 3D Printing

A composite material consisted of a PLA matrix (Ingeo 2003D, Natureworks, Blair, NE, USA) with 30 wt.% Lyocell fibres (FCP fibres with norminal length of 0.3 mm, supplied by Lenzing, Lenzing an der Ager, Austria) was used as an active/responsive layer in the bi-material structures. First, the polymer and fibres were melt compounded using a Process 16 Parallel Twin-Screw Extruder, (Thermo Fisher Scientific Inc., Waltham, MA, USA), processed with a temperature profile of 165 °C, 180 °C, and the rest 185 °C at 70 rpm. The obtained material was pelletised and used for the production of 3D printing filaments similarly to what has been previously done [63]. The pelletised composite (<4 mm particles) was processed in a Filabot EX2 single screw extruder at 180 °C. The filament was air-cooled and spooled in a custom-made spooling device synchronised with the extrusion speed to obtain filaments of 1.75 ± 0.1 mm. For the passive layers, only PLA was used. The PLA filament was produced using a 3Devo filament maker and at a temperature ranging from 175–190 °C (Feed to die).

The two different types of material were thoroughly dried in a vacuum oven at 45 °C overnight (a period of at least 12 h) before being printed. The printing was performed using a Voron 350 FDM printer fitted with a 0.8 mm nozzle. The filament was printed at 220 °C onto a 60 °C bed. The layer height was set at 0.2 mm, and the part was printed at 20 mm/s. 

Two types of design were used. A rectangular shape was adopted to explore three layouts of active-to-passive layers, while assemblies with a triangular shape were used to visualise opening and closing in a scaled-down assembly. In both rectangular and triangle designs, the first layer consisting of the composite active material was printed with 100% infill at raster angle of 90° and without perimeter. The second layer of PLA only was printed with a 50% infill and with a raster angle of 0°. A perimeter was printed for a stronger outline. Table 3 shows the slicer settings for the 3D-printed composites. The rigid support frame that holds the panels together within the assembly, was 3D printed using a commercially available filament (Guen, ColorFabb, Belfeld, The Netherlands). 

The different layouts were used to assess their influence on the shape-change characteristics of the bi-material strips/triangles. A passive-to-active layer ratio of 1:1 is commonly used for hygromorphic structures, whereas, for PLA-lyocell composites, a lower ratio of passive to active layers has been shown to produce a more pronounced shape change or curvature [64]. In this research, the aim was to evaluate whether thicker samples (layout 2) would exhibit similar transformations to thinner samples (layout 1) by altering the passive-to-active layer ratio. Layout 3 was defined after initial trials with the other layouts revealed excessive curvature at the tips of the samples. 

#### 2.2.2. Layer Variation Based on Rectangular Shape 

**Figure 5:**
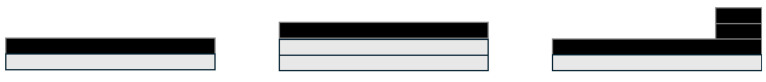
Schematic side view of the layout 1 (**left**), layout 2 (**middle**), layout 3 (**right**). Black represents the passive layer; grey represents the active composite layer.

Rectangular shapes were 3D printed to quantitively evaluate the deformation and compare three different layouts (Figure 5). The rectangles were 80 × 10 mm in length and width (with thickness based on the layout), and five samples of each design were printed and tested at once.

a. Layout 1: One active layer and one passive layer (total thickness of 0.4 mm),
b. Layout 2: Two active layers and one passive layer (total thickness of 0.6 mm),
c. Layout 3: One active layer and one passive layer with 2 extra passive layers of 100% infill at one end that were 20 mm in length.

The environmental experiment on the samples was conducted at room temperature. These conditions were

a. Water spray from a spray bottle (until thoroughly wet) to activate the shape change transformation;
b. Monitoring/drying for 24 h at 23 °C and 50% RH;
c. Drying at 40 °C oven for 1 h.

**Figure 6:**
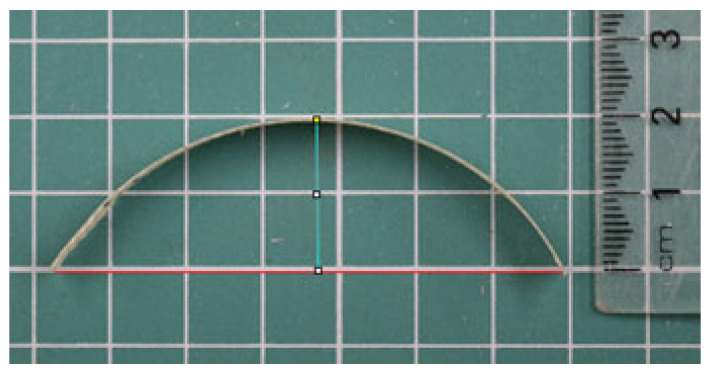
Base (chord length) and height lines drawn in ImageJ for radius calculation.

The samples were allowed to move freely without any mechanical constraints. Photographs were taken at 0.1, 1, 2, 4, and 24 h to measure the height and radius of curvature. For each photograph, the sample was positioned on its side, and the tip height was measured using a ruler (Figure 6). The radius of curvature was determined by drawing base and height lines in ImageJ (Version 1.54p) 1.54 m and applying a macro based on the chord length formula [63].

K = 8d/(c^2^ + 4d^2^) (1)

where K is curvature in mm^−1^, d is the height in mm, and c is the chord length in mm. 

The chord method is a well-established approach to approximate curvature, particularly in contexts where arc measurements may be impractical or less precise [28,63].

#### 2.2.3. Assemblies (Triangular Shapes)

**Figure 7:**
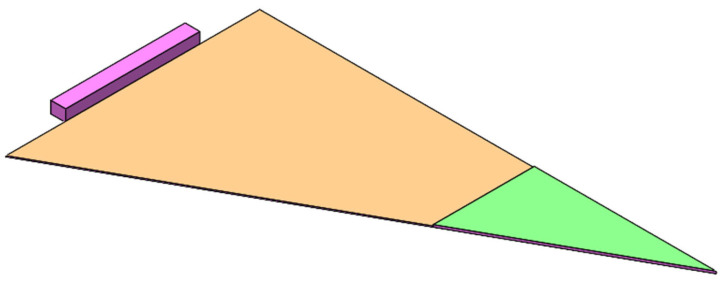
Triangular assembly 2 based on Layout 3. Further modified composite (orange) triangle including a tab for assembly (purple) and reinforced tip (green).

The triangle assemblies were composed of four 3D printed intermeshing triangles. Two assembly layouts were tested based on Layout 1 and 3 of the rectangular shapes. The thicker section of Layout 3 was added to the narrowest part of the triangle (Figure 7). A tab was also added to the triangle shape to the slit onto the overall frame. This thicker section was made of extra layers of PLA. Glue was used to fix the triangles assemblies into the slits of the frame. 

The triangular structure performances were tested under the following the same environmental conditions as the rectangular samples:

a. Water spray from a spray bottle (until thoroughly wet at room temperature) to activate the shape change transformation,
b. Monitoring/drying for 24 h at 23 °C and 50% RH,
c. Drying at 40 °C oven for 1 h

Photographs were taken throughout the process to monitor the shape variation.

## 3. Results 

### 3.1. Motorised Shading System with Mechanical Actuation (3D Printing)

A comparative analysis of the nine developed kinematic shading systems was conducted based on six key evaluation criteria: Simplicity and Robustness, Actuation Method, Repairability, Mounting Feasibility, Occlusion Ratio, and Architectural Integration (Table 4). From this analysis, three designs—Rotating Plates, Simplified Origami Fold, and Overlapping Slats—were shortlisted for further refinement, based on their balance of mechanical performance, scalability, and integration potential. 

Similar qualitative comparative analysis have been applied in the evaluation of kinetic facades and deployable structures [60].

(1) **Rotating Plates** performs strongly across all criteria with minimal compromises. Mechanically simple, visually clean, and architecturally feasible—making it a strong candidate for real-world application and scaling.
  - **Simplicity & Robustness**: Rated high—uses few moving parts, reducing potential failure points.
  - **Actuation**: Dual servos offer reliable, precise control with relatively simple implementation.
  - **Repairability**: Rated high—shutters are removable, allowing easy maintenance or replacement.
  - **Mounting Feasibility**: Rated high—design integrates easily with minimal impact on the building structure.
  - **Occlusion Ratio**: Rated high—capable of full closure, supporting effective shading.
(2) **Simplified Origami Fold** offers a good trade-off between mechanical novelty and practical deployment. It’s visually interesting, functionally adaptable, and easier to manufacture than the original fold.
  - **Simplicity & Robustness**: Rated high—fewer hinges than the original origami design means greater reliability and easier fabrication.
  - **Actuation**: Linear actuator is straightforward and compatible with many control systems.
  - **Repairability**: Rated moderate—simple geometry aids maintenance, though less accessible than Rotating Plates.
  - **Mounting Feasibility**: Rated good—modular and scalable; fits well into façade grid systems.
  - **Occlusion Ratio**: Moderate—lower than the original origami fold, but still functional.
(3) **Overlapping Slats** Despite not achieving full occlusion, it offers dynamic surface texture and shading performance with simpler construction than more complex biomimetic or multi-layered options. Its aesthetic appeal in modular repetition adds architectural value.
  - **Simplicity & Robustness**: Moderate—requires precise alignment, but manageable.
  - **Actuation**: Uses dual servos—reliable and compact.
  - **Repairability**: Moderate—complexity in alignment may pose some maintenance challenges.
  - **Mounting Feasibility**: Moderate—integration into a grid is possible with thoughtful planning.
  - **Occlusion Ratio**: Low—does not achieve full opacity, but performs well for typical solar angles.

The three shortlisted concepts—Rotating Plates, Simplified Origami Fold, and Overlapping Slats—were prototyped at a 1:5 scale to assess their mechanical feasibility and design integration.

Although testing was conducted at reduced scale, consideration was given to the challenges of full-scale implementation to ensure the most appropriate concept was selected. The Simplified Origami Fold demonstrated potential for compact deployment and minimal spatial footprint. However, its fold-and-slide mechanism proved complex to fabricate, even at 1:5 scale. Issues with hinge alignment and actuator integration highlighted concerns regarding scalability and reliability. Despite these challenges, its flexibility and spatial efficiency remain promising for 1:1 deployment, provided further refinement is undertaken. In contrast, Rotating Plates and Overlapping Slats were mechanically more straightforward. Their rotation-based mechanisms involved fewer components, simplifying fabrication and improving reliability. While these designs lacked the formal complexity and visual appeal of the Origami concept, their simplicity, robustness, and ease of maintenance position them as strong candidates for practical application in adaptive façade systems (Table 5).

Rotating plates were chosen because they offer a simple rotational mechanism that is easy to fabricate and provides good structural stability. Unlike overlapping slats, which can occupy more space when open and may show gaps when closed, rotating plates present no significant challenges and also allow for the creation of various patterns.

The Rotating Plates concept was selected for physical prototyping at a 1:5 scale to evaluate mechanical feasibility, modular integration, and readiness for upscaling. The prototype array was constructed using 2020 aluminium extrusion as the structural frame, with 3D-printed servo mounts and SG90 micro servos to actuate individual shutters.

**Figure 8:**
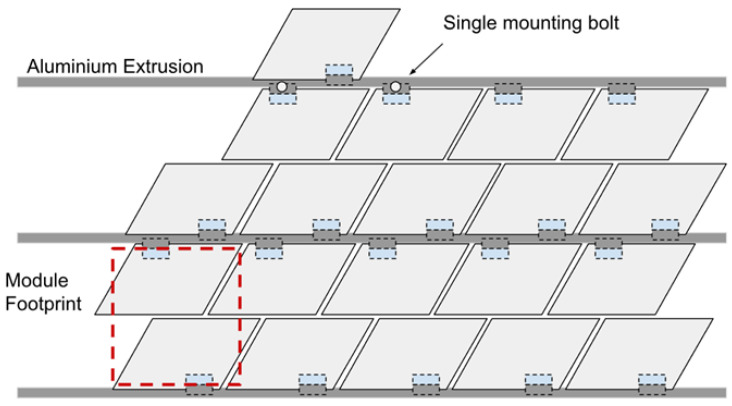
Design of the 1:5 assembly.

**Figure 9:**
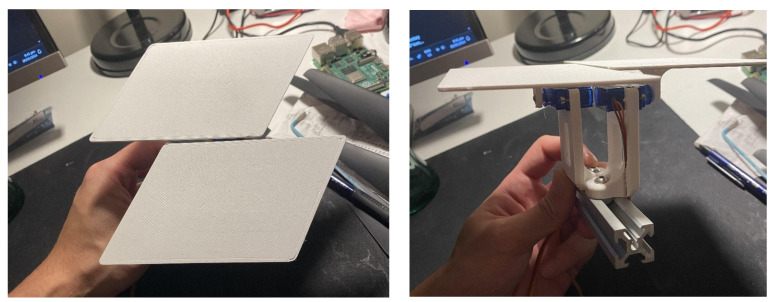
Top and bottom shutters from above view (**Left**) and side view attached to the frame (**Right**).

Each module consisted of a pair of rotating plates mounted to a shared aluminium rail using a single bolt (Figure 8), allowing for rotational adjustment and ease of alignment. The design enabled the top shutter of a lower module and the bottom shutter of the upper module to share a mounting rail, reducing overall material use and visual obstruction. The extrusion framework also served as a routing channel for servo wiring, with grooves integrated into the mounts for cable concealment (Figure 9). 

**Figure 10:**
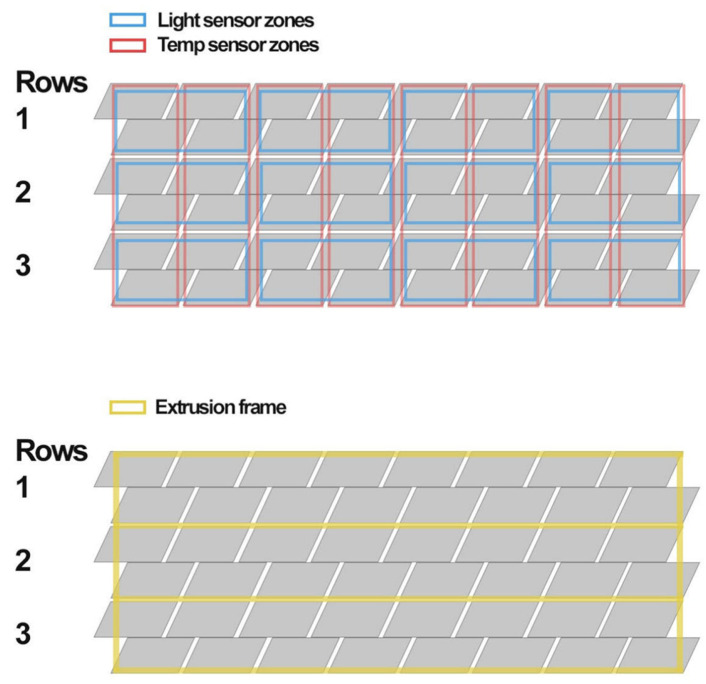
Design of sensor placement (**Top**) and support frame (**Bottom**).

Sensor zones were mapped across the façade and included temperature and light sensors to simulate future feedback-based control but were not installed (Figure 10 Top). The servo mounts were designed to support lateral adjustment and to conceal servo wiring within integrated grooves, enhancing visual clarity and protecting cables during operation (Figure 10 Bottom). The SG90 servos proved sufficient for low-load actuation at this scale, but limitations were evident, including mechanical noise, restricted torque capacity, and a minimum step size of approximately one degree. These constraints could pose challenges for precision and durability in a full-scale deployment. 

**Figure 11:**
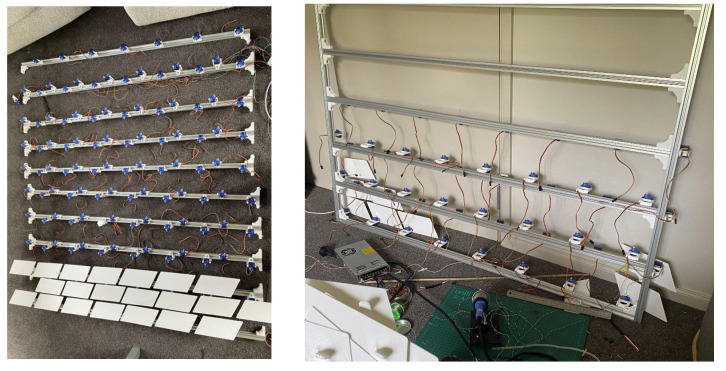
Scale prototype of the Rotating Plates concept being built, arrays (**left**) and arrays mounted onto a vertical frame (**right**).

The façade prototype was fabricated with the following dimensions: 675 cm (W) × 400 cm (D) × 250 cm (H), scaled down to 1:5. A complete module fitted a 160 mm square footprint with a 10mm gap around its perimeter. Additionally, the fixed distance between the horizontal frames required the shutter mounts to ensure sufficient clearance between actuated shutters to prevent interference. The complete 1:5 façade prototype (Figure 11) consisted of 27 servo-controlled modules. Each was pre-mounted to vertical aluminium rails before being installed on the frame. This modular approach allowed for efficient installation and wiring. The mounting strategy included a single bolt per shutter, which enabled small rotational adjustments during alignment. Each module occupied a footprint of 160 mm by 160 mm, with a 10 mm buffer zone to prevent interference between rotating parts. The system demonstrated strong repeatability and structural stability, validating the underlying mechanical concept.

At 1:5 scale, SG90 micro servos provide sufficient torque to rotate lightweight 3D-printed panels. However, in a full-scale (1:1) system, the mass, inertia, and wind load on each panel would increase significantly. The transition from model to full scale will require re-evaluation of actuation power, mechanical durability, and environmental resilience. The elegant simplicity of the rotating mechanism remains valid, but would be paired with industrial-grade components, stronger materials, and more sophisticated control systems.

### 3.2. Non-Motorised Shading Systems with Passive Actuation (4D Printing)

#### 3.2.1. Rectangular Shape Characterisation 

**Figure 12:**
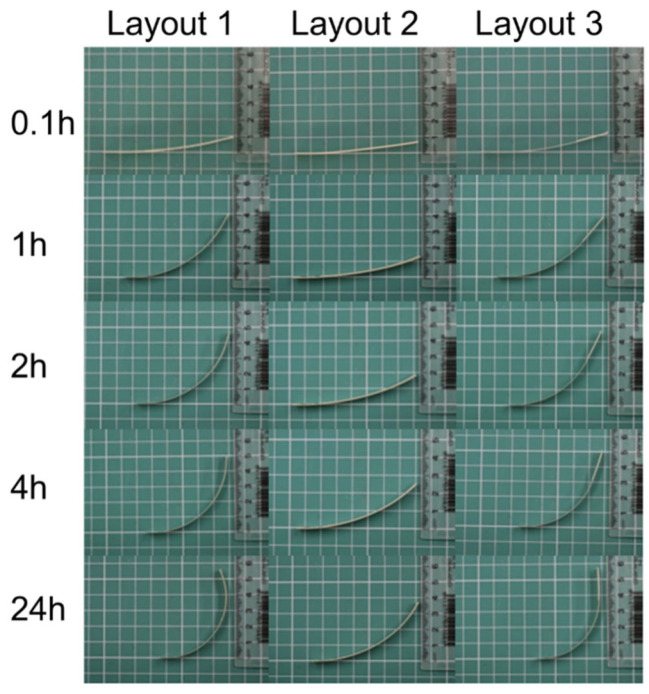
Photographs of the three rectangular layouts over time displaying deformation due to moisture.

As reported by others, both the actuation amplitude and response rate are influenced by the geometry and material composition [28,63]. Layouts 1 and 3 showed similar height variation with asymmetrical curving; however, the asymmetrical reinforcement in Layout 3 prevented over-curling on one side of the sample (Figure 12). In contrast, Layout 2—with a double active layer—exhibited a reduction in movement despite the increased volume of actuation material. This behaviour can be attributed to an approximate 50% increase in overall thickness, which markedly enhances bending stiffness, given that flexural rigidity scales with the cube of the section thickness [65].

Layout 1 with one passive layer and one active layer measured approximately 0.340 mm, while the layout 2 with two active layers and one passive layer measured around 0.570 mm. Layout 3 had the same thickness of layout 1 but with and extra 0.340 mm of passive material at the end of the samples.

**Figure 13:**
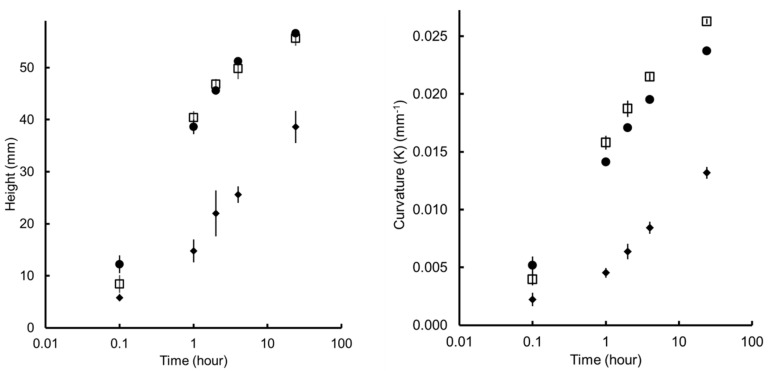
Variation in the height (left) and the curvature (right) as a function of time for Layout 1 (Square), Layout 2 (Black diamond) and Layout 3 (Black circle). The error bar represents the standard deviation for n = 5. The figure does include error bars; however, the errors are so small that they are contained within the size of the marker.

When plotting the measurement variation as a function of time, Layout 2 (thickest samples) exhibited the slowest response and produced curvatures approximately 30% smaller than those observed for Layouts 1 and 3 (Figure 13). In all layouts, the fastest deformation occurs within the first hour and slowed down within 24 h. Layouts 1 and 3 showed no noticeable difference in height performance; however, reinforcing one end of the sample altered the curvature profile and resulted in an approximate 10% decrease in K. This prevented the “over-curling” effect observed in Layout 1. 

The plots indicate that all three layouts exhibit rapid actuation within the first hour of exposure. In both height and curvature measurements, a steep increase is observed between 0.1 and 1 h, highlighting that most of the shape change occurs within this early window. This suggests that the material–geometry system responds quickly to moisture ingress. Notably, the Layout 1 (circle) and Layout 3 (square) achieve over 80% of their final curvature and displacement before the 4-h mark, with only marginal changes, thereafter, indicating a fast approach to equilibrium. In contrast, the sample represented by Layout 2 (diamond) displays slower kinetics and lower overall actuation, with a more gradual increase in both height and curvature over time. This is expected as Layout 2 is thicker, and water diffusion is expected to be slower.

#### 3.2.2. First Assembly

**Figure 14:**
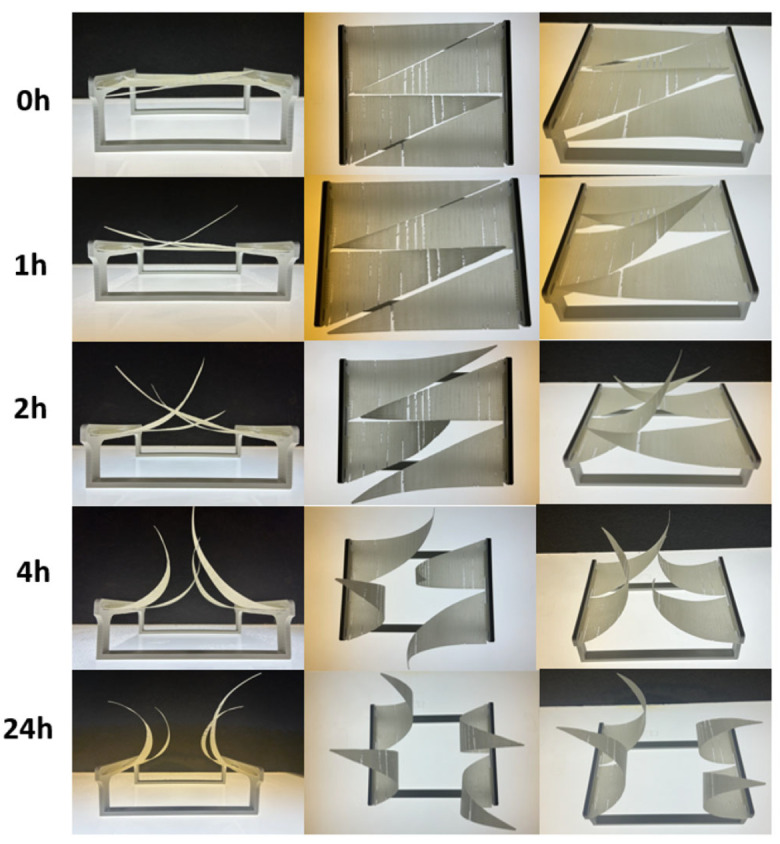
Evolution of the Assembly One over time under moisture stimulus.

The passive testing of the triangle relied on fixing one end to a frame in cantilever mode and observing the curvature as moisture entered the composite, with photos taken at 1, 2, 4, and 24 h (Figure 14). The stiffness of the prints was reduced by the presence of random holes, which still provided partial shading but also decreased structural integrity. Similar to the rectangular-shaped composites, the triangular prints appeared to “over-curl” within 24 h, indicating that the movement was too pronounced. Some twisting was also observed, particularly toward the shorter leg of the triangle. This torsional behaviour is likely due to geometric and fabrication-related asymmetries. The triangular layout, with one side fixed and uneven leg lengths, creates an imbalance in stiffness that promotes twisting during activation. Additionally, the anisotropic properties inherent in the FDM printing process—especially differences in raster orientation between the triangle’s edges—may add to uneven strain distribution. Residual stresses from printing, such as those caused by uneven cooling or layer deposition, may also relax over time and contribute to the observed out-of-plane twisting. While the primary goal is bending-driven actuation, this torsional response adds complexity to the motion and need to be accounted for in future design refinements. 

#### 3.2.3. Second Assembly 

**Figure 15:**
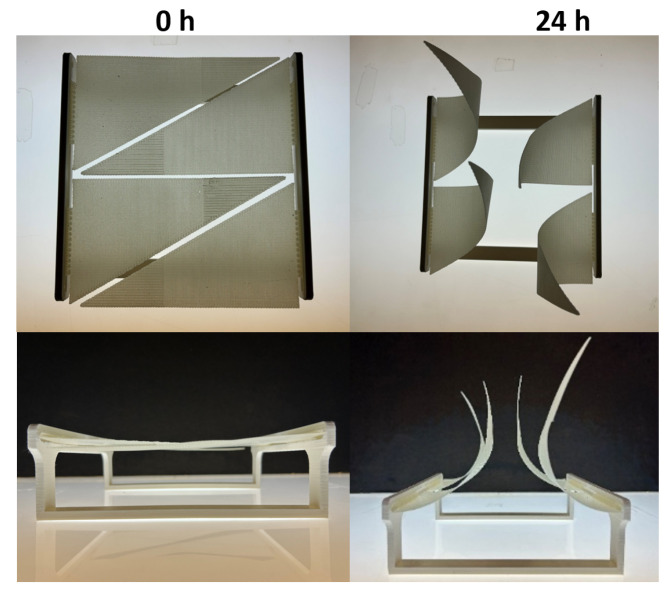
Second assembly of the structure in closed and opened positions.

Four triangles were 3D printed using the same design with the exception of the tip reinforcement section that was increased in length from 25% of total length of the triangles (Figure 15). The slicer settings were the same as Table 3, with the addition of 2 passive layers with 100% infill at the tip. These composite triangles were then glued into the frame, sprayed with water and left for 24 h at ambient conditions. The reinforcement at the apex (pointed tip) locally increased stiffness and altered the overall deformation pattern. As expected, the reinforced tip resisted curling, leading to an obvious reduction in curvature compared to the unreinforced layout of the first assembly. This was consistent with a measured decrease in the curvature coefficient (K) by approximately 10% in the rectangular samples. Interestingly, while the overall bending was less pronounced, the sample exhibited more pronounced torsional behaviour. The deformation was accentuated compared to the first assembly toward the shorter edge of the triangle. This suggests that the interaction between the fixed base, the asymmetric geometry, and the local reinforcement creates an uneven stress distribution during actuation that was exacerbated by an increase in stiffness. As the reinforced tip constrains movement at one end, the free edges—especially the shorter leg—become the dominant paths for curvature and twist. The result is a less uniform, more complex motion that combines bending and asymmetric torsion, highlighting the sensitivity of the system to even minor stiffness modifications.

## 4. Discussions and Conclusions

The comparative evaluation and 1:5 scale prototyping revealed the trade-offs between mechanical simplicity, fabrication ease, and architectural performance in kinetic façade systems. While visually expressive designs like the Simplified Origami Fold offered compactness and aesthetic potential, their complexity in actuation and fabrication raised concerns about scalability and long-term reliability. In contrast, the Rotating Plates and Overlapping Slats concepts, though less visually complex, demonstrated mechanical robustness and practical feasibility, attributes critical for deployment at architectural scale.

The selection of the Rotating Plates concept for physical prototyping underscored the importance of modularity, ease of integration, and structural clarity in kinetic shading systems. Although limitations in small-scale components such as servo torque and sensor implementation were identified, the overall mechanical concept proved sound.

This project also demonstrated in parallel the feasibility of using hygromorphic composite materials to create passive structures capable of dynamic shape change in response to environmental humidity. Specifically, bi-layered PLA/Lyocell composites, printed in combination with pure PLA, showed strong and consistent bending behaviour when exposed to moisture, confirming their suitability for adaptive applications. 

Different materials have been used in hygromorphic systems. Wood-based approaches rely on differences in hygroexpansion between laminates with different grain orientations [66]. Although highly responsive, they face limitations in uniformity, manufacturability, and durability. Other studies use natural-fibre yarns or short fibres in a polymer matrix, opening up additional manufacturing routes, including additive manufacturing, which enables effective fibre alignment in predefined orientations.

Lyocell fibres are advantageous due to their uniform dimensions and predictable mechanical properties, enhancing processability and facilitating 3D printing of biocomposites. They also exhibit strong hygroscopic behaviour. In PLA composites, comparable moisture uptake yields ~3% hygroexpansion for lyocell versus ~0.7% for wood fibre [50,64]. Even relative to composites with 70 wt% continuous flax fibres in a PP matrix [67], which achieved a maximum hygroexpansion of ~3.4% in the transverse direction, the ~3% observed for lyocell is comparatively high. In addition, using short fibres rather than continuous fibres offers broader manufacturing options. Together, these factors suggest a higher potential for shape-change/curvature than systems based on other fibres.

The triangular actuator design successfully produced curvature and height changes over time, and partial design optimisation—such as tip reinforcement and multi-layer configurations—helped evaluate the influence of stiffness distribution and geometry. However, several opportunities for further refinement remain. Replacing the triangular shape with a rectangular profile may reduce unwanted torsion and improve packing density by allowing closer spacing and better shading coverage. Extending the composite length to create slight overlap across the frame could further increase functional coverage. Adjustments to the stiffened tip—either by increasing its area or removing active material entirely—could better control the onset and magnitude of curling, ensuring that the actuation reaches its target state at the desired time.

The PLA-based formulations were primarily used for research and prototyping purposes. It is important to mention the limitations of PLA due to its low UV resistance and biodegradability, which would restrict its use in real-life applications. However, its high moisture absorption is not considered a drawback in this context, since it is essential for the system to function, the composites must absorb water and expand for the shape transformation to occur. The main aim of this study was to demonstrate the concept and produce a prototype using the selected design. In practical applications, other stable and durable polymers could be used with lyocell fibres, such as polyketone (PK) or high-density polyethylene (HDPE), with the addition of UV stabilisers. 

This study on hygromorphic composite materials has certain limitations that warrant acknowledgment. First, the cyclability of the developed materials was not examined in detail. While preliminary investigations were performed, this aspect was not pursued further, as it lies beyond the scope of the present work. Future studies should address long-term performance and repeatability, which are critical for practical applications.

Another important consideration relates to thickness effects. It is well established that increasing thickness raises bending stiffness, which in turn slows the hygromorphic response and water migration within the samples. Prior studies [64,66] have shown that both the passive-to-active layer ratio and the overall thickness of bilayer hygromorphic structures govern curvature. This behavior can be described using a modified Timoshenko equation, originally developed for bimetallic strips with differing thermal expansion coefficients and later adapted for differential hygroexpansion. Such relationships provide a means to tune both the magnitude and rate of transformation. Although highly relevant to the potential applications of the structures presented here, this line of inquiry is outside the scope of the current manuscript and represents a promising direction for future investigation.

In addition, the materials used in this study were selected primarily for proof-of-concept purposes. While the incorporation of hygromorphic materials demonstrates feasibility, the scalability of 3D printing with PLA/lyocell remains uncertain. Factors such as the manufacturing process and the overall thickness of the structures may influence performance, including the risk of delamination. These challenges underscore the need for further research into alternative material systems and process optimization before practical deployment can be realized.

The development of 3D-printed motorised shading systems highlighted several technical and practical challenges that will need to be addressed in future stages. These include improving sensor integration, refining control and communication systems, selecting appropriate materials and fabrication methods, and developing effective mounting strategies for both new and existing buildings. Considerations around scalability, system reliability, and ease of installation will be key to ensuring the viability of the design in real-world architectural applications.

Future work should focus on refining material design and actuation strategies to balance responsiveness, stability, and durability, while also standardising testing methods for consistent evaluation across studies. To enable real-world application in adaptive façades, challenges related to manufacturability, long-term performance, and modelling of moisture–structure interactions must be addressed through advanced optimisation and predictive tools.

## Figures and Tables

**Table 2:** Synthesis of 3D printing materials/processes findings in passive actuation drawn by past studies.

Material	Stimuli	Technical Characteristics	Variable	Method of Fabrication	Transition Time	Application	Authors
**PLA**	Temperature (Hot water at 80 °C) Bending	Tg: 60~65 °C, Tm: 180 °C	Geometric shapes & patterns, Stored strain (by Print parameters; feed rate and stretching of filament, print temperature & direction)	Fused deposition moulding (FDM)printer	Not mentioned	Soft robotics, grippers & self-morphing structures	[46]
**Chitosan hydrogel ink & Pretrained polystyrene (PS) film**	Heat absorbed through light at 3.5 megawatts per mm Bending	Tg of shrink film is 105 °C	spatial pattern	Ink 3D printed on film then dried at 50 °C	30 to 60 s	Responsive truss structure	[42]
**Hydrogel-based ink**	Moisture and temperature Bending	Temperature above 32 °C, tensile strength: 642 KPa	Polymer chains in the ink formulations	3D printed as ink then UV polymerised	Immediate transition	Biomedical applications	[47]
**PLA Filament**	Temperature at 105 °C Bending	Elastic modulus: 50 MPa, Tg: 60~65 °C	Thickness Stored stress during printing, released upon heating.	FDM 3D printer.	18 s	Engineering applications such as filtering, localising and guiding acoustic waves, shape-changing solar concentrators	[48]
**Soybean oil epoxidized acrylate**	Temperature Fixed temporary shape at −18 °C, recovered original shape at human body temperature (37 °C)	Tg: 37 °C, UV curable	printer infill density, laser frequency and printing speed.	Custom-developed multilateral 3D printer system, laser printing technique	1 min	Biomedical scaffolds	[49]
**Wood-fill fine filament (includes PLA, PHA & wood fibres)**	Moisture	Young’s modulus:—0° compressed composite: ~4000 MPa with ~0.15% swelling—90° compressed composite: ~3500 MPa with ~0.43% swelling—90° high-speed printing: ~900 MPa with ~0.5% swelling		Prusa I3 Rework 3D printer equipped with a 0.4 mm nozzle	1 day to 1 h	Passive devices for solar tracking systems, responsive building skins	[50]
***Bacillus subtilis* natto cell**	Moisture	50% swelling. Resists up to 2 min flush of water, 470° curvature		Inkjet 3D printer	A few seconds	Adaptive interface and wearable applications	[51]
**Polyester wood filled filament and ABS**	Moisture	Mutli-phase motion based on direction of prints	Geometric shapes & patterns,	FDM	Min- to hours	Not specifically mentioned	[52]
**Wood-fill (15, 25%) filament (PLA and arbocel)**	Moisture	Degree and direction of bending depend on the PLA-to-wood-PLA layer ratio		FDM			[53]
**Wood-fill (40%) PLA filament (Laywood)**	Moisture and temperature	Cantilever actuator achieved a curvature of 120° at 120 min and progressed to 270° at 270 min under controlled humidity conditions	Porosity/infill, patterns, layers	FDM	Min-hours	Speculative: soft robotics, adaptive architecture, and responsive textiles,	[54]

**Table 3:** General slicer settings for the 3D printed composites both triangular and rectangular shapes.

Parameters	Slicer Settings
Line thickness [mm]	0.9
Layer height [mm]	0.2
Print speed [mm/s]	20
Nozzle temperature [°C]	220
Build plate temperature [°C]	60
Active density [%]	100
Active raster angle [°]	90
Passive density [%]	50
Passive raster angle [°]	0

**Table 4:** Comparative evaluation of nine kinematic shading design concepts.

Design Concept	Simplicity & Robustness	Actuation Method	Repairability	Mounting Feasibility	Occlusion Ratio
**Circular Shutters**	Moderate—simple geometry but required depth for retraction	Servo or motor-geared	Moderate—moving parts enclosed in the frame	Limited—requires depth for installation on the facade	Moderate—partial occlusion
**Origami Fold**	Low—complex with many hinges	Linear actuator or motor-linkages	Low—intricate parts are harder to access	Complex—required rail system	High—near full closure
**Folding Wing**	High—simple folding with few parts	Linear actuator	High—accessible and modifiable	High—minimal structural impact	Moderate—needed tuning
**Rotating Plates**	High—few moving parts	Dual servos	High—removable shutters	High—minimal structural impact	High—full occlusion is achievable
**Overlapping Slats**	Moderate—phasing required precise alignment	Dual servos	Moderate—Posing complexity for maintenance	Moderate—required careful planning	Low—not fully opaque
**Sequential Layered Fold**	Moderate—cascading motion increased complexity	Dual servos	Low—difficult to access, maintain and repair	Moderate—required stable support	High—multi-stage occlusion
**Simplified Origami Fold**	High: reduced hinges, improved reliability	Linear actuator	Moderate—minimal complexity	Good—modular and scalable	Moderate—less occlusion than the original origami
**Scissored Mechanism Slats**	Moderate—known mechanism but spatially inefficient	Linear actuator	Moderate—layout, components and spatial arrangement lack efficiency and are hard to maintain and integrate.	Poor—large footprint even when compressed	Low—gaps remained in a closed state
**Expanding Wing**	Low—intricate linkages prone to failure	Gears and linkages	Low—difficult to service reliably	Low—challenging to integrate	Moderate—smoother transitions

**Table 5:** Key strengths and challenges of the three shortlisted shading concepts based on 1:5 scale prototyping.

Design	Strengths	Challenges
**Simplified Origami Fold**	Minimal spatial footprint	Complex hinging and alignment requirements Difficult to integrate actuators at prototype scale (1:5) The design at a scale of 1:1 could be compromised by the increased thickness of materials A more complex fold and slide mechanism
**Overlapping Slats**	Simple rotational mechanism Ease of fabrication Reliable and mechanically robust	Occupies more space when open, as both wings cover the length of the frame Gaps may be visible even if fully closed
**Rotating Plates**	Simple rotational mechanism Ease of fabrication Good structural stability Potential for creating a variety of patterns	No significant challenge was observed compared to the other two designs

## Data Availability

The datasets presented in this article are not readily available because the data are part of an ongoing study. Requests to access the datasets should be directed to corresponding author.

## Abbreviations

The following abbreviations are used in this manuscript:

PLA	Polylactic acid
FDM	Fused Deposition Modelling
Bio-ABFs	Biomimetic Adaptive Building Façades
SMAs	Shape memory alloys

## References

[1] IEA (2025). Energy Efficiency Policy Toolkit 2025.

[2] Globalabc (2023). IEA Tracking Report—Buildings. https://globalabc.org/index.php/resources/publications/iea-tracking-report-buildings.

[3] Dewidar K., Mahmoud A.H., Magdy N., Ahmed S. The role of intelligent façades in energy conservation. Proceedings of the International Conference on Sustainability and the Future: Future Intermediate Sustainable Cities (FISC 2010).

[4] Francesco S., Lidia B., Gigliola A. (2022). A critical review of biomimetic building envelopes: Towards a bio-adaptive model from nature to architecture. Renew. Sustain. Energy Rev..

[5] Imani N., Vale B. (2021). Heating with Wolves, Cooling with Cacti: Thermo-Bio-Architectural Framework (ThBA).

[6] Attia S., Lioure R., Declaude Q. (2020). Future trends and main concepts of adaptive facade systems. Energy Sci. Eng..

[7] Al-Masrani S.M., Al-Obaidi K.M. (2019). Dynamic shading systems: A review of design parameters, platforms and evaluation strategies. Autom. Constr..

[8] Hosseini S.M., Mohammadi M., Guerra-Santin O. (2019). Interactive kinetic façade: Improving visual comfort based on dynamic daylight and occupant’s positions by 2D and 3D shape changes. Build. Environ..

[9] Imani N., Vale B. (2022). Biomimetic Design for Building Energy Efficiency 2021. Biomimetics.

[10] Charpentier L., Cruz E., Nenov T., Guidoux K., Ware S. (2022). Pho’liage: Towards a kinetic biomimetic thermoregulating façade. Bionics and Sustainable Design.

[11] Imani N., Vale B. (2022). Biomimetic Buildings: Copying Nature for Energy Efficiency.

[12] Meagher M. (2015). Designing for change: The poetic potential of responsive architecture. Front. Archit. Res..

[13] Alemdağ E.L., Beyhan F. (2017). A research on construction systems of double skin facades. Gazi Univ. J. Sci..

[14] Schumacher M., Schaeffer O., Vogt M.-M. (2012). Move: Architecture in Motion-Dynamic Components and Elements.

[15] Sheikh W.T., Asghar Q. (2019). Adaptive biomimetic facades: Enhancing energy efficiency of highly glazed buildings. Front. Archit. Res..

[16] Hosseini S.M., Mohammadi M., Rosemann A., Schröder T., Lichtenberg J. (2019). A morphological approach for kinetic façade design process to improve visual and thermal comfort. Build. Environ..

[17] Körner A., Born L., Mader A., Sachse R., Saffarian S., Westermeier A., Poppinga S., Bischoff M., Gresser G., Milwich M. (2017). Flectofold—A biomimetic compliant shading device for complex free form facades. Smart Mater. Struct..

[18] Badarnah L., Zolotovsky K. (2022). Morphological differentiation for the environmental adaptation of biomimetic buildings: Skins, surfaces, and structures. Biomimicry for Materials, Design and Habitats.

[19] López M., Rubio R., Martín S., Croxford B. (2017). How plants inspire façades. From plants to architecture: Biomimetic principles for the development of adaptive architectural envelopes. Renew. Sustain. Energy Rev..

[20] Mi-jin K., Baek-gyeom K., Je-sung K., Hwang Y. (2023). Flexural biomimetic responsive building façade using a hybrid soft robot actuator and fabric membrane. Autom. Constr..

[21] Saidam M.W., Al-Obaidi K.M., Hussein H., Ismail M.A. (2017). The application of smart materials in building facades. Ecol. Environ. Conserv..

[22] Holstov A., Farmer G., Bridgens B. (2017). Sustainable materialisation of responsive architecture. Sustainability.

[23] Smith S.I. (2017). Superporous intelligent hydrogels for environmentally adaptive building skins. MRS Adv..

[24] Villegas J.E., Gutierrez J.C.R., Colorado H.A. (2020). Active materials for adaptive building envelopes: A review. J. Mater. Environ. Sci..

[25] Yi H., Kim D., Kim Y., Kim D., Koh J.-S., Kim M.-J. (2020). 3D-printed attachable kinetic shading device with alternate actuation: Use of shape-memory alloy (SMA) for climate-adaptive responsive architecture. Autom. Constr..

[26] Reichert S., Menges A., Correa D. (2015). Meteorosensitive architecture: Biomimetic building skins based on materially embedded and hygroscopically enabled responsiveness. Comput. Aided Des..

[27] Imani N., Donn M., Balador Z. (2018). Bio-inspired materials: Contribution of biology to energy efficiency of buildings. Handbook of Ecomaterials.

[28] de Kergariou C., Demoly F., Perriman A., Le Duigou A., Scarpa F. (2023). The design of 4D-printed hygromorphs: State-of-the-art and future challenges. Adv. Funct. Mater..

[29] Barbier M., Le Guen M.J., McDonald-Wharry J., Bridson J.H., Pickering K.L. (2021). Quantifying the shape memory performance of a three-dimensional-printed biobased polyester/cellulose composite material. 3D Print. Addit. Manuf..

[30] Khezri M., Rasmussen K. (2022). Functionalising buckling for structural morphing in kinetic façades: Concepts, strategies and applications. Thin-Walled Struct..

[31] Talaei M., Mahdavinejad M., Azari R., Haghighi H.M., Atashdast A. (2022). Thermal and energy performance of a user-responsive microalgae bioreactive façade for climate adaptability. Sustain. Energy Technol. Assess..

[32] Schumacher M., Vogt M.-M., Krumme L.A.C. (2019). New Move: Architecture in Motion-New Dynamic Components and Elements.

[33] ArchDaily One Ocean, Thematic Pavilion EXPO 2012/Soma. https://www.archdaily.com/236979/one-ocean-thematic-pavilion-expo-2012-soma.

[34] Schleicher S., Lienhard J., Poppinga S., Speck T., Knippers J. (2015). A methodology for transferring principles of plant movements to elastic systems in architecture. Comput. Aided Des..

[35] Krieg O.D. (2016). HygroSkin—Meteorosensitive Pavilion. Advancing Wood Architecture.

[36] Attia S. (2017). Evaluation of adaptive facades: The case study of Al Bahr Towers in the UAE. QScience Connect..

[37] Glynn R., Sheil B. (2017). Fabricate 2011: Making Digital Architecture.

[38] Loonen R.C., Rico-Martinez J., Favoino F., Brzezicki M., Ménézo C., La Ferla G., Aelenei L.L. (2015). Design for façade adaptability: Towards a unified and systematic characterization. Proceedings of the 10th Conference on Advanced Building Skins.

[39] Wigginton M. (2002). Intelligent Skins. Butterworth-Heinemann Linacre House.

[40] Cheng T., Tahouni Y., Sahin E.S., Ulrich K., Lajewski S., Bonten C., Wood D., Rühe J., Speck T., Menges A. (2024). Weather-responsive adaptive shading through biobased and bioinspired hygromorphic 4D-printing. Nat. Commun..

[41] Koh J.-S., Kim S.-R., Cho K.-J. (2014). Self-folding origami using torsion shape memory alloy wire actuators. International Design Engineering Technical Conferences and Computers and Information in Engineering Conference.

[42] Zolfagharian A., Kaynak A., Khoo S.Y., Kouzani A. (2018). Pattern-driven 4D printing. Sens. Actuators A Phys..

[43] Sung D. (2016). Smart geometries for smart materials: Taming thermobimetals to behave. J. Archit. Educ..

[44] Foged P. (2016). Sense Envelope IV. https://pasoldfoged.com/sense-iv.

[45] Mazzucchelli E.S., Alston M., Brzezicki M., Doniacovo L. (2018). Study of a BIPV adaptive system: Combining timber and photovoltaic technologies. J. Facade Des. Eng..

[46] Alshebly Y.S., Mustapha K.B., Zolfagharian A., Bodaghi M., Mohamed Ali M.S., Almurib H.A., Nafea M. (2022). Bioinspired pattern-driven single-material 4D printing for self-morphing actuators. Sustainability.

[47] Naficy S., Gately R., Gorkin III R., Xin H., Spinks G.M. (2017). 4D printing of reversible shape morphing hydrogel structures. Macromol. Mater. Eng..

[48] Zhang Q., Zhang K., Hu G. (2016). Smart three-dimensional lightweight structure triggered from a thin composite sheet via 3D printing technique. Sci. Rep..

[49] Miao S., Zhu W., Castro N.J., Nowicki M., Zhou X., Cui H., Fisher J.P., Zhang L.G. (2016). 4D printing smart biomedical scaffolds with novel soybean oil epoxidized acrylate. Sci. Rep..

[50] Le Duigou A., Castro M., Bevan R., Martin N. (2016). 3D printing of wood fibre biocomposites: From mechanical to actuation functionality. Mater. Des..

[51] Yao L., Ou J., Cheng C.-Y., Steiner H., Wang W., Wang G., Ishii H. BioLogic: Natto cells as nanoactuators for shape changing interfaces. Proceedings of the 33rd Annual ACM Conference on Human Factors in Computing Systems.

[52] Correa D., Poppinga S., Mylo M.D., Westermeier A.S., Bruchmann B., Menges A., Speck T. (2020). 4D pine scale: Biomimetic 4D printed autonomous scale and flap structures capable of multi-phase movement. Philos. Trans. R. Soc. A.

[53] Krapež Tomec D., Straže A., Haider A., Kariž M. (2021). Hygromorphic Response Dynamics of 3D-Printed Wood-PLA Composite Bilayer Actuators. Polymers.

[54] Tahouni Y., Krüger F., Poppinga S., Wood D., Pfaff M., Rühe J., Speck T., Menges A. (2021). Programming sequential motion steps in 4D-printed hygromorphs by architected mesostructure and differential hygro-responsiveness. Bioinspiration Biomim..

[55] Eppinger S.D., Ulrich K. (1995). Product Design and Development.

[56] Mangkuto R.A., Koerniawan M.D., Apriliyanthi S.R., Lubis I.H., Atthaillah, Hensen J.L., Paramita B. (2021). Design optimisation of fixed and adaptive shading devices on four façade orientations of a high-rise office building in the tropics. Buildings.

[57] Al Dakheel J., Tabet Aoul K. (2017). Building Applications, opportunities and challenges of active shading systems: A state-of-the-art review. Energies.

[58] Roshan Kharrat R. (2024). Dynamic and Adaptive Photovoltaic Shading Systems: Design and Architectural Integration for Energy Production and Indoor Comfort. Ph.D. Thesis.

[59] Tabadkani A., Haddadi M., Rizi R.A., Tabadkani E. (2023). A hierarchical multi-purpose roller shade controller to enhance indoor comfort and energy efficiency. Building Simulation.

[60] Konstantoglou M., Tsangrassoulis A. (2016). Dynamic operation of daylighting and shading systems: A literature review. Renew. Sustain. Energy Rev..

[61] Wu H., Zhang T. (2022). Optimal design of complex dynamic shadings: Towards sustainable built environment. Sustain. Cities Soc..

[62] Brzezicki M. (2024). A Systematic Review of the Most Recent Concepts in Kinetic Shading Systems with a Focus on Biomimetics: A Motion/Deformation Analysis. Sustainability.

[63] Gauss C., Pickering K., Barbier M., Miller T. (2023). Additive manufacturing of hygromorphic structures using regenerated cellulose/PLA biocomposites. Mater. Today Proc..

[64] Bachmann P.A., Pickering K., Gauss C. (2025). Combining Hydromorphic and Shape-Shifting Effects for Programmable Passive Actuation in 4D-Printed Biocomposites. Compos. Part. C Open Access.

[65] Timoshenko S. (1925). Analysis of bi-metal thermostats. J. Opt. Soc. Am..

[66] Holstov A., Bridgens B., Farmer G. (2015). Hygromorphic materials for sustainable responsive architecture. Constr. Build. Mater..

[67] Le Duigou A., Keryvin V., Beaugrand J., Pernes M., Scarpa F., Castro M. (2019). Humidity responsive actuation of bioinspired hygromorph biocomposites (HBC) for adaptive structures. Compos. Part. A Appl. Sci. Manuf..

